# Genetics of supraventricular tachycardia: current evidence with a focus on translational relevance and personalized medicine

**DOI:** 10.3389/fcvm.2026.1832643

**Published:** 2026-05-29

**Authors:** Amin Esmailian, Mohammad Alasti

**Affiliations:** 1 Department of Medicine, Peninsula University Hospital, Frankston, Victoria, Australia; 2 Department of Cardiology, Victorian Heart Hospital, Clayton, Melbourne, Victoria, Australia; 3 Department of Cardiology, Victorian Heart Institute, Monash University, Clayton, Melbourne, Victoria, Australia

**Keywords:** AVNRT, AVRT, cardiogenetics, genetics, inherited arrhythmia, personalized-medicine, supraventricular tachycardia

## Abstract

**Background:**

Supraventricular tachycardias (SVTs) are among the most common arrhythmias encountered in clinical practice and, despite generally low mortality, impose substantial morbidity and healthcare utilization. Clinical heterogeneity in age of onset, recurrence, symptom burden, and overlap with conduction disease or cardiomyopathy suggests underlying biological determinants, including inherited susceptibility. Over the past decade, advances in human genomics have expanded evidence from familial aggregation and rare syndromic disorders to population-scale genome-wide association studies (GWAS), but routine translation into SVT care remains limited.

**Methods:**

Narrative review of guideline-based SVT definitions and mechanistic frameworks, familial and rare-variant studies, GWAS/meta-analyses across SVT subtypes (AVNRT, accessory pathway-mediated AVRT/WPW, and focal atrial tachycardia), and translational literature on biomarkers (including microRNA/exosomal biology), functional validation models, and implementation considerations (yield, cost-effectiveness, ethics, and governance)

**Results:**

SVT demonstrates subtype-specific genetic architecture. AVNRT is supported by familial clustering and polygenic/oligogenic susceptibility, with GWAS signals implicating developmental and myocardial structural pathways (e.g., NKX2-5, TTN, MYH6). Accessory pathway-mediated AVRT/WPW shows the clearest genotype-to-substrate relationship, with common and rare variation implicating conduction and junctional developmental biology (including SCN5A/SCN10A- and CCDC141-linked signals, and emerging family-based discoveries such as MRC2). In contrast, the genetic basis of focal atrial tachycardia remains underpowered and mechanistically heterogeneous. The highest current clinical utility of genetics lies in identifying syndromic and cardiomyopathy-associated SVT (e.g., PRKAG2, LAMP2), where diagnosis alters prognosis, surveillance, and cascade screening.

**Conclusion:**

Genetic discoveries are reshaping SVT from a purely “functional” arrhythmia toward a spectrum of inherited electrophysiologic and myocardial disease. While routine genetic testing is not indicated for most isolated SVT, phenotype-guided evaluation, improved functional models, and implementation frameworks may enable targeted, patient-centred personalization particularly in early-onset, familial, or cardiomyopathy-overlap presentations.

## 1 Introduction

Supraventricular tachycardias (SVTs) represent a diverse group of cardiac arrhythmias characterized by rapid heart rhythms originating above the His-Purkinje system. SVT are commonly encountered in the clinical practice and account for a substantial proportion of emergency department presentations, outpatient electrophysiology referrals, and catheter ablation procedures worldwide (1). Although often considered benign from a mortality perspective, SVTs are associated with significant morbidity, including recurrent palpitations, syncope, impaired exercise tolerance, anxiety, reduced quality of life, and, in selected cases, tachycardia-mediated cardiomyopathy (2, 3). Contemporary clinical cohorts further demonstrate substantial healthcare utilization, particularly in patients with recurrent arrythmia burden or coexisting structural or genetic heart disease substrates (4–8).

Historically, the diagnosis and management of SVTs have relied on electrocardiographic documentation, ambulatory rhythm monitoring, invasive electrophysiological (EP) mapping, and an understanding of re-entrant and triggered arrhythmia mechanisms (1–3). Catheter ablation has transformed outcomes for many SVT subtypes, achieving high acute success and low recurrence rates in experienced centers (1). However, even in the era of highly effective procedural therapy, considerable heterogeneity persists in clinical presentation, age at onset, recurrence risk, arrhythmia burden, response to pharmacological therapy, and coexistence with structural or cardiomyopathic phenotypes. These observations suggest that SVT susceptibility and expression are not solely stochastic phenomena but are influenced by underlying biological determinants, including genetic factors and their interaction with autonomic and environmental modifiers. Even in apparently “isolated” SVT phenotypes, latent cardiomyopathy or conduction system disease may emerge over time, supporting a broader view of SVT within myocardial and conduction system biology (9–12).

Over the past decade, advances in human genetics and genomics have begun to illuminate the inherited contributions to SVT. Evidence now spans familial aggregation studies, twin and heritability analyses, rare monogenic syndromes, and population-based genome-wide association studies identifying common susceptibility variants across SVT subtypes (11, 13, 14). Parallel progress in next-generation sequencing, bioinformatics, and functional genomics has accelerated gene discovery and expanded opportunities to link genetic variation with electrophysiological mechanisms. Conceptually, SVT genetics lies on a spectrum from high-impact rare variants underlying syndromic and familial disease to polygenic susceptibility in common electrophysiologically defined phenotypes. Despite these advances, translation into routine clinical care remains limited (14–16). Barriers include incomplete phenotype–genotype correlation, modest effect sizes for common variants, challenges in functional validation of functional validation of conduction system substrates, and uncertainty regarding clinical utility and cost-effectiveness (14, 15, 17). Implementation-focused work in inherited arrhythmia and cardiogenetics clinics provides a pragmatic template for SVT genetics translation, emphasizing phenotype-first evaluation, targeted testing strategies, rigorous variant interpretation, and multidisciplinary governance (15, 17, 18).

This narrative review synthesizes current evidence on the genetic predictors of SVT with an explicit focus on translational relevance and personalized medicine. We integrate molecular, clinical, and implementation perspectives to address how genetic discoveries can refine SVT risk stratification, inform therapeutic decision-making, and ultimately bridge the gap between mechanistic insight and patient-centred care. In addition to gene discovery, we highlight emerging translational concepts including polygenic risk approaches, bioinformatic and machine-learning enabled variant prioritization, and pharmacogenomic considerations that may influence drug exposure and tolerability even when SVT-specific genotype-guided therapy is not yet established.

To ensure comprehensive coverage, the evidence base incorporated in this narrative includes electrophysiology-guideline definitions and mechanistic frameworks (1, 16), Atrio-Ventricular Nodal Re-entrant Tachycardia (AVNRT)-focused familial and genomic studies (11, 13, 19), accessory pathway and Wolf-Parkinson-White (WPW) genomic and familial risk evidence (12, 20, 21), syndromic and cardiomyopathy-overlap genetics (22, 23), and mechanistic or translational molecular work including microRNA, exosomal biology, and signalling pathways relevant to atrial and supraventricular substrates (24–28).

## 2 Definitions and classification of supraventricular tachycardia

A precise definition of SVT is essential for genetic investigation, as mechanistic heterogeneity directly influences the interpretation of genetic associations. According to contemporary European Society of Cardiology (ESC) guidelines, SVT encompasses tachyarrhythmias originating above the His-bundle and includes a spectrum of atrial and atrioventricular junctional arrhythmias (1, 16). Clinically, SVTs most commonly present as regular narrow-complex tachycardias, defined by a QRS duration ≤120 ms, although wide-complex presentations may occur due to pre-existing bundle branch block, aberrant conduction, or conduction over an accessory pathway, including antidromic Atrio-Ventricular Re-entrant Tachycardia (AVRT) (1). The subtypes most relevant to genetic inquiry include AVNRT, AVRT mediated by an accessory pathway, and atrial tachycardias, most commonly focal atrial tachycardia (FAT).

AVNRT is the most prevalent SVT in adults, accounting for approximately 60% of cases, with a higher incidence in women, and is characterized by re-entry within or adjacent to the atrioventricular node facilitated by functionally distinct fast and slow pathways with differing conduction velocities and refractory properties (1). In contemporary genetic studies, AVNRT is typically defined as a regular, narrow-QRS tachycardia involving atrioventricular nodal tissue, with diagnosis confirmed by invasive electrophysiological study demonstrating dual AV nodal physiology and inducible nodal re-entry, thereby minimizing phenotypic misclassification. AVRT arises when an accessory pathway provides an alternative atrioventricular conduction route, enabling macro-re-entrant circuits incorporating both the atrioventricular node–His system and the accessory pathway, or incorporating two different accessory pathways. Manifest pathways produce ventricular pre-excitation, classically recognized as WPW syndrome, whereas concealed pathways conduct only retrogradely and lack baseline pre-excitation (1, 29, 30). Atrial tachycardias represent a more heterogeneous group and include FAT driven by abnormal automaticity, triggered activity, or micro-re-entry, as well as macro-re-entrant atrial tachycardias defined by larger re-entrant circuits (31).

Atrial flutter represents a prototypical macro-re-entrant atrial tachycardia and therefore falls within the broader SVT framework, but it is frequently analysed separately in genetic studies owing to its unique substrate and clinical behaviour. Accordingly, exclusion of AF and atrial flutter from SVT-focused genetic analyses is necessary to avoid conflating biologically disparate arrhythmic mechanisms.

Likewise, the term paroxysmal SVT describes a clinical presentation of abrupt-onset tachycardia rather than a specific electrophysiological mechanism and may encompass AVNRT, AVRT, or FAT (1, 16). While clinically pragmatic, paroxysmal SVT represents a genetically imprecise phenotype unless supported by electrophysiological subtyping. This definitional precision is essential when interpreting studies that include mixed supraventricular phenotypes and when contextualizing SVT genetics within the broader biology of atrial and conduction system disease (1, 32).

Against this framework of rigorously defined SVT phenotypes, recent genetic studies have begun to delineate subtype-specific susceptibility loci, providing insight into the molecular architecture underlying atrioventricular nodal and atrial conduction system arrhythmogenesis.

## 3 Pathophysiology of supraventricular tachycardia

### 3.1 Atrioventricular nodal re-entrant tachycardia (AVNRT)

AVNRT arises from the specialised micro-anatomy and electrophysiological heterogeneity of the atrioventricular (AV) junction. Rather than a discrete anatomical structure, the AV node comprises a three-dimensional transitional region extending from the atrial myocardium into the compact node and His bundle, characterised by regional variation in conduction velocity, refractory properties, intercellular coupling, and autonomic innervation (1, 16, 33). These intrinsic gradients give rise to functional dual-pathway physiology, classically described as a fast pathway with relatively rapid conduction and longer refractory period and a slow pathway with slower conduction and shorter refractoriness. The inducibility and stability of this circuit depend on inter-individual differences in nodal tissue architecture, conduction velocity gradients, and refractory properties, rather than a discrete anatomical re-entry pathway.

At a mechanistic level, these nodal properties are shaped by molecular pathways governing cardiac conduction system development, myocyte differentiation, and electrical coupling. Genes implicated in AVNRT susceptibility encode regulators of conduction system patterning (NKX2-5), atrial cardiomyocyte structure and excitability (MYH6), and sarcomeric integrity with downstream effects on mechano-electrical signalling (TTN) (13). Variation within these pathways may influence ion channel distribution, connexin expression, and cellular alignment within the AV junction, exaggerating physiological dissociation between fast and slow pathways and lowering the threshold for re-entry.

Autonomic modulation further interacts with this substrate. Parasympathetic activation preferentially prolongs fast-pathway refractoriness, whereas sympathetic stimulation enhances conduction velocity and shortens refractoriness, dynamically altering pathway balance and facilitating re-entrant activation under permissive conditions (1, 16, 33). AVNRT can therefore be understood as a disorder of functional AV nodal organisation, arising from the interaction of genetically influenced nodal architecture, electrophysiological properties, and autonomic tone even in the absence of overt structural heart disease (13, 19, 29, 34).

### 3.2 Atrioventricular re-entrant tachycardia and accessory pathways

AVRT and WPW syndrome arise from abnormalities in cardiac development affecting atrioventricular electrical insulation. During normal embryogenesis, progressive formation of the fibrous atrioventricular annulus electrically separates atrial and ventricular myocardium, leaving the atrioventricular node–His bundle as the sole conduction pathway between chambers. Incomplete formation of this insulating tissue results in persistence of muscular atrioventricular connections, termed accessory pathways, which can conduct electrical impulses between atria and ventricles (1). The electrophysiological properties of accessory pathways including conduction velocity, effective refractory period, and the presence or absence of decremental conduction determines the clinical and arrhythmic phenotype (1, 29).

Manifest pathways conduct anterogradely and produce ventricular pre-excitation on surface electrocardiography, whereas concealed pathways conduct only retrogradely and remain electrically silent during sinus rhythm. In AVRT, re-entry occurs when the atrioventricular node-His-Purkinje system and the accessory pathway form a macro-re-entrant circuit, most commonly with anterograde conduction through the atrioventricular node and retrograde conduction via the accessory pathway (orthodromic AVRT), or less commonly the reverse (antidromic AVRT) (1, 33, 34).

Because accessory pathways represent persistent structural remnants of atrioventricular myocardial continuity, AVRT constitutes one of the most developmentally grounded and genetically interpretable forms of supraventricular tachycardia. Mechanistically, susceptibility is likely influenced by genetic variation affecting myocardial patterning, cell-cell adhesion, extracellular matrix composition, and formation of the atrioventricular annulus, processes essential for normal electrical insulation during cardiac morphogenesis (35–37).

This developmental model is supported by familial aggregation studies demonstrating inherited susceptibility to accessory pathways and WPW syndrome, as well as population-based genetic analyses implicating loci near genes involved in cardiac development and conduction system biology (12, 20). Collectively, these observations support a model of AVRT as a disorder of aberrant atrioventricular myocardial persistence, in which genetically influenced developmental variation permits formation of accessory conduction pathways that subsequently enable re-entrant tachycardia in structurally normal hearts.

Less common accessory pathway variants should also be acknowledged within the spectrum of SVT-causing anatomic substrates. These include atriofascicular, nodofascicular, nodoventricular, and fasciculoventricular pathways, often grouped historically under the term “Mahaim’ pathways (38–41). In contrast to typical atrioventricular accessory pathways, Mahaim-type pathways characteristically show anterograde decremental conduction and are most often right-sided, with atriofascicular connections representing the best-described subtype. Their clinical relevance lies not only in their ability to sustain antidromic or other pre-excitation-related tachycardias, but also in the diagnostic and procedural challenges they pose. Fasciculoventricular pathways are generally regarded as bystander pathways rather than integral limbs of re-entrant circuits, making recognition important to avoid unnecessary or potentially harmful ablation near the AV node or His bundle. Similarly, nodofascicular and nodoventricular pathways may require especially careful mapping because successful ablation sites can be close to the slow pathway region, proximal conduction system, or right bundle area (38–41).

### 3.3 Focal atrial tachycardia (FAT)

FAT is mechanistically distinct from re-entrant supraventricular tachycardias and arises from localized sources of abnormal impulse initiation rather than sustained macro-re-entry. The predominant mechanisms include enhanced automaticity and triggered activity mediated by delayed afterdepolarizations, with micro-re-entry playing a lesser role (1, 3, 31). These arrhythmias commonly originate from reproducible atrial regions such as the crista terminalis, tricuspid annulus, coronary sinus ostium, and pulmonary vein region, reflecting regional heterogeneity in atrial electrophysiological properties (42, 43).

The biological substrate underlying FAT is more heterogeneous than that of AVNRT or AVRT. Abnormalities in intracellular calcium handling, adrenergic signalling, ion channel regulation, and localized atrial microstructure can promote spontaneous depolarization or triggered activity without a fixed re-entrant circuit (31, 44).

In paediatric populations, FAT is more frequently associated with congenital heart disease or cardiomyopathy, suggesting a stronger developmental and genetic contribution in selected cases (45). However, the scarcity of large, electrophysiologically well-characterized cohorts has limited genetic investigation of FAT, representing a key knowledge gap (1, 43).

## 4 Genetic architecture of SVT

SVT is increasingly supported as a genetically influenced trait with subtype-specific biology, shaped by common polygenic susceptibility and an additional contribution from rare variants in biologically plausible pathways. At the population level, genome-wide association studies (GWAS) provide the most direct evidence that common variants contribute to SVT risk. For AVNRT, the first dedicated GWAS identified association signals near NKX2-5 and sarcomeric genes including TTN and MYH6 (lead variants reported include rs10071514 near NKX2-5, rs2562834 in TTN, and rs365990 in MYH6) (13). A subsequent multi-ancestry GWAS meta-analysis confirmed subtype-specific architecture, identifying genome-wide significant loci for AVNRT at NKX2-5 (rs6882776) and TTN (rs4471922), and for accessory pathway-mediated AVRT at SCN5A (rs9843500), an independent signal at SCN10A (rs10428132), and a TTN/CCDC141 locus (rs7573293) (14).

This meta-analysis also identified additional suggestive SVT loci relevant to mechanistic heterogeneity and future replication. For AVNRT, suggestive associations were observed at or near PRRX1, DPF3, CTSB, ACTN1, and PVALB/NCF4, while AVRT/accessory pathway analyses identified suggestive signals near ANKRD28, NEDD9, and ST8SIA5/PIAS2 (14). These signals are notable because they cluster in pathways plausibly relevant to atrial and nodal biology, including cytoskeletal organization, chromatin regulation, and neural or migratory programs that may influence arrhythmia susceptibility.

These loci map to coherent mechanisms relevant to SVT pathophysiology. NKX2-5 encodes a cardiac transcription factor essential for cardiac morphogenesis and specification and maintenance of specialized conduction tissue, providing a plausible developmental route to altered AV nodal architecture and re-entry susceptibility (46, 47). SCN5A encodes the principal cardiac sodium channel NaV1.5, a key determinant of excitability and conduction velocity in working myocardium and specialized conduction tissue (48, 49). The accessory pathway literature provides particularly clear common-variant examples: a GWAS of accessory atrioventricular pathways identified three independent coding risk variants, two in CCDC141 (p.Arg935Trp and p.Ala141Val) and one in SCN10A (p.Ala1073Val), with all three variants associating with PSVT, and p.Ala1073Val also associating with AF. The same alleles were associated with higher heart rate and shorter P-R interval, linking genotype to measurable conduction and chronotropic traits (20). Mechanistic interpretation was supported by evidence that the SCN10A signal may act via effects on nearby SCN5A expression and sodium-channel mediated conduction (20).

The involvement of TTN and MYH6 supports a second mechanistic axis, namely cardiomyocyte structural and electromechanical properties. Titin and sarcomeric variation can influence myocardial architecture and mechano-electric signalling that modulates excitability and conduction stability, particularly in atrial and nodal regions predisposed to triggered activity or re-entry (13).

Complementing GWAS, rare-variant approaches provide early evidence that non-syndromic SVT is enriched for variation in pathways affecting conduction and tissue organization, although discovery remains limited by cohort size and phenotype heterogeneity. Whole-exome sequencing in AVNRT has nominated candidate genes spanning ion channels and conduction biology and pathways related to neuronal or neurotransmitter signalling, supporting interaction between nodal electrophysiology and autonomic modulation (50).

Family-based genetics has also advanced mechanistic gene discovery for re-entrant SVT with WPW-pattern phenotypes: a 2024 study identified a rare nonsynonymous variant in MRC2 in pedigrees with re-entrant SVT and WPW ECG pattern, supported by functional work in a knock-in mouse model showing increased re-entrant SVT and bypass tract formation despite preserved cardiac structure (21). These findings support a model in which common variation establishes a baseline susceptibility, while rarer variants in development, conduction, and myocardial remodelling pathways contribute to familial clustering and extreme phenotypes.

SVT genetics lies on a continuum with inherited cardiomyopathies and storage disorders, in which supraventricular arrhythmias, pre-excitation, and conduction disease can be prominent. PRKAG2 syndrome is a classic example, with ventricular pre-excitation and supraventricular arrhythmias alongside progressive conduction disease and cardiomyopathic remodelling (12, 22). Danon disease due to LAMP2 variants is another archetype in which cardiomyopathy and characteristic ECG abnormalities may include WPW-pattern or pre-excitation, and arrhythmic complications contribute substantially to outcomes (23).

**Figure 1:**
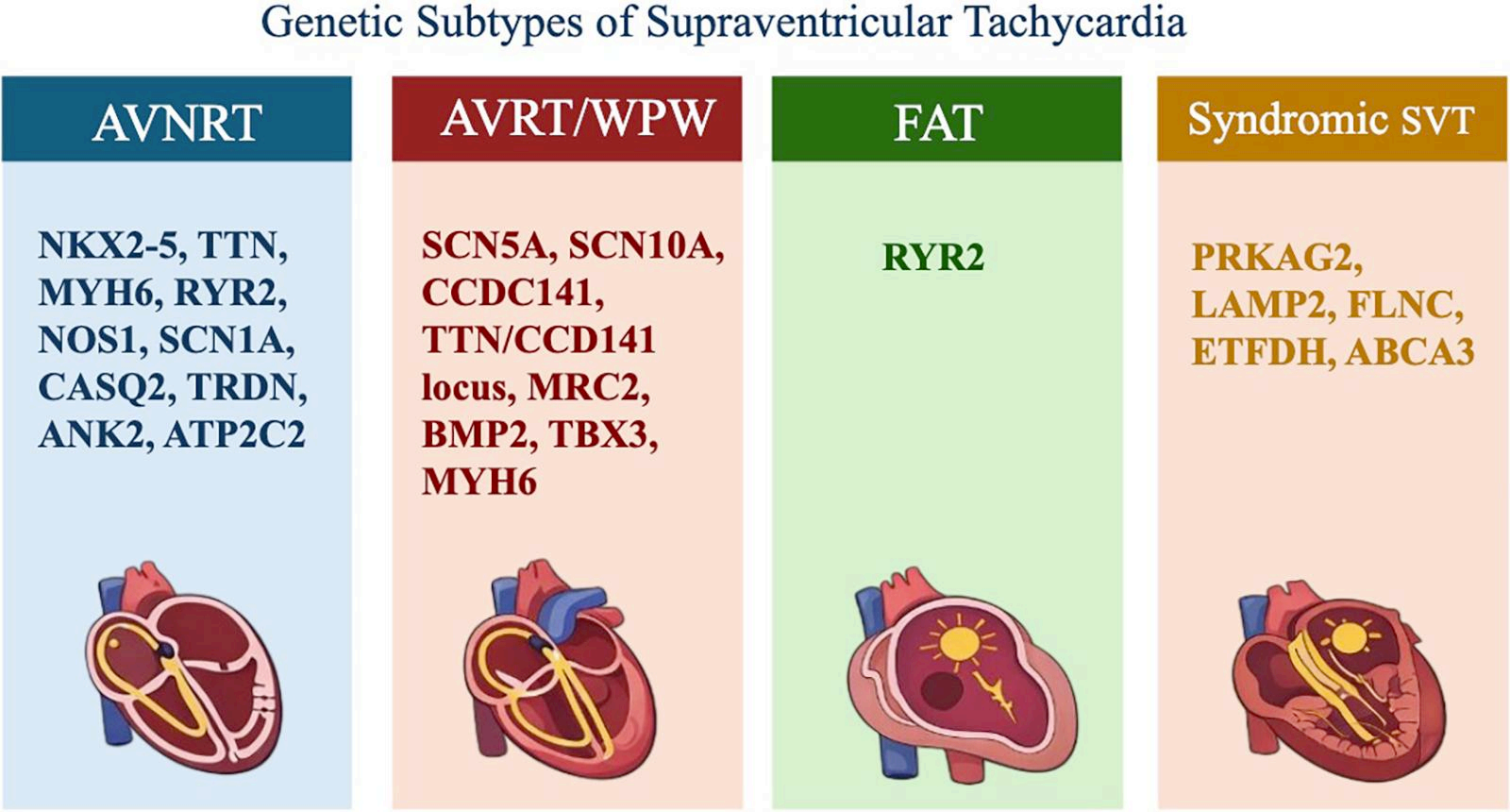
Overview of subtype-specific genetic associations in supraventricular tachycardia.

The above subtype-specific genetic architecture is summarised in Table 1 and Figure 1.

**Table 1:** Subtype-specific genetic architecture of supraventricular tachycardia.

SVT subtype	Main electrophysiologic substrate	Nature of genetic evidence	Key genes/loci implicated	Proposed biological mechanism	Current translational relevance
AVNRT	Functional dual AV nodal physiology with re-entry within/adjacent to the AV node	Familial clustering, GWAS, rare-variant sequencing, pathway-based WES studies	NKX2-5, TTN, MYH6; candidate pathway genes include RYR2, NOS1, SCN1A, CASQ2, TRDN, ANK2, ATP2C2	Developmental patterning of conduction tissue, myocardial structural/electromechanical effects, calcium handling, autonomic modulation	Primarily mechanistic at present; routine genetic testing is generally low yield in isolated adult-onset AVNRT
Accessory Pathway-mediated AVRT/WPW	Persistent muscular atrioventricular connections enabling macro-re-entry	Strongest subtype-specific evidence: GWAS, familial studies, rare variants, structural variation	SCN5A, SCN10A, CCDC141, TTN/CCDC141 locus, MRC2; additional candidates BMP2, TBX3, MYH6	Abnormal atrioventricular insulation, conduction biology, extracellular matrix/cell-matrix interactions, developmental patterning of AV junction	Most genetically interpretable non-syndromic SVT; supports targeted evaluation in early-onset, familial, pre-excitation, or cardiomyopathy-overlap cases
Focal atrial tachycardia (FAT)	Localized abnormal impulse initiation: automaticity, triggered activity, or micro-re-entry	Sparse evidence; small familial and paediatric series; no robust large-scale locus discovery yet	No reproducible FAT-specific loci; indirect signal from RYR2 in selected early-onset/paediatric cases	Calcium-handling dysfunction, adrenergic signalling, atrial microstructural heterogeneity	Remains underpowered and heterogeneous; presently research-focused rather than clinically actionable
Syndromic/cardiomyopathy-associated SVT	SVT occurring as part of a broader myocardial, metabolic, or conduction disorder	Monogenic/syndromic evidence with direct clinical relevance	PRKAG2, LAMP2, FLNC; rarer examples include ETFDH, ABCA3	Glycogen storage/metabolic remodelling, lysosomal dysfunction, structural cardiomyopathy, progressive conduction disease	Highest current clinical utility of genetics in SVT; diagnosis changes prognosis, surveillance, and family screening
Inappropriate sinus tachycardia (IST)	Enhanced sinus node automaticity and autonomic dysregulation	Very limited; rare familial/channelopathy report	No established loci; rare HCN4 gain-of-function variants	Increased pacemaker current (If), *β*-adrenergic hypersensitivity, impaired parasympathetic modulation	No current role for genetic testing; primarily clinical diagnosis

## 5 Subtype-specific genetics of supraventricular tachycardia

### 5.1 Genetic determinants of AVRNT

AVNRT has long been framed as a largely functional re-entrant arrhythmia arising from dual AV nodal physiology. However, converging clinical and genomic evidence supports an inherited contribution to the AV nodal substrate that permits inducible re-entry. Familial clustering has been repeatedly documented, ranging from early multi-kindred series to larger electrophysiology-based cohorts. In the earliest systematic family descriptions, dual AV nodal physiology and inducible AVNRT were demonstrable in most evaluated affected relatives, supporting a heritable predisposition to the dual-pathway substrate (10).

In a larger ablation cohort study, familial AVNRT occurred more often than previously recognized (prevalence 127 per 10,000), and first-degree relatives had a substantially increased likelihood of AVNRT compared with the general population, consistent with autosomal dominant inheritance with incomplete penetrance (11). A systematic case-series review similarly noted that most familial cases represent typical (slow-fast) AVNRT, reinforcing that the classic phenotype segregates within families rather than an unusual AVNRT variant (51).

Population-scale genomics has now provided direct evidence of common-variant susceptibility. The first AVNRT GWAS identified three independent association signals near NKX2-5 and within TTN and MYH6 (including rs10071514 near NKX2-5, rs2562834 in TTN, rs365990 in MYH6) (13). Notably, the same study also reported a significant genetic correlation between AVNRT and AF, indicating partial overlap in inherited susceptibility despite distinct clinical phenotypes (13).

A subsequent multi-ancestry GWAS meta-analysis further supported NKX2-5 and TTN as AVNRT loci and added functional triangulation via transcriptome-wide association, in which reduced predicted NKX2-5 expression in cardiac tissues was associated with increased AVNRT risk (14). These findings highlight developmental and myocardial structural pathways rather than a single channelopathy mechanism.

Rare-variant studies in EP-confirmed cohorts suggest additional heterogeneity and point to pathways that can plausibly modulate nodal refractoriness and triggered atrial inputs that initiate re-entry. Targeted next-generation sequencing across 67 genes in nearly 300 AVNRT patients identified numerous rare variants distributed across sodium-handling, calcium-handling, and HCN channel genes, with many individuals carrying variants affecting sodium and calcium handling. However, no single gene class showed definitive rare-variant enrichment, consistent with a burden-type architecture rather than a monogenic pattern (19).

Whole-exome sequencing studies have further highlighted pathway-level signals, nominating candidate genes spanning calcium cycling and excitation-contraction coupling (for example RYR2, NOS1), ion-channel biology (for example SCN1A), and neuronal/neurotransmitter-release pathways, suggesting that AVNRT susceptibility may reflect combined effects on nodal electrophysiology and autonomic modulation (50). In familial AVNRT pedigrees, integrative WES analyses converged on a calcium-signalling module (including CASQ2, TRDN, RYR2, NOS1, ANK2, ATP2C2) as a plausible mechanistic axis for inherited susceptibility (52).

From a translational standpoint, AVNRT genetics currently has limited direct impact on routine clinical decision-making for typical adult-onset AVNRT. Its primary value is mechanistic, demonstrating that inherited variation can shape the developmental and electrophysiologic substrate and explain familial aggregation and variable age of onset, even in the absence of overt structural heart disease (10, 11, 13, 14).

### 5.2 Genetics of AVRT and WPW syndrome

Accessory pathway–mediated AVRT and WPW syndrome provide one of the clearest genotype-to-mechanism relationships in supraventricular tachycardia, as the underlying substrate reflects a developmental failure of atrioventricular electrical insulation. Persistence of muscular atrioventricular connections across the annulus fibrosus results in a fixed anatomical pathway for re-entry, making AVRT a paradigmatic developmental arrhythmia.

GWAS provide compelling evidence that common genetic variation contributes to accessory pathway formation. A large GWAS of individuals with accessory atrioventricular pathways identified genome-wide significant coding variants in CCDC141 (p.Arg935Trp and p.Ala141Val) and SCN10A (p.Ala1073Val), with the same alleles also associating with paroxysmal SVT, consistent with AVRT representing the dominant clinical manifestation of accessory pathways (20). A subsequent multi-ancestry GWAS meta-analysis further implicated loci mapping to SCN5A, SCN10A, and a TTN/CCDC141 region in AVRT and accessory pathway phenotypes, reinforcing a dual mechanistic axis involving sodium channel–mediated conduction properties and developmental susceptibility of the atrioventricular junction (14). Together, these findings establish accessory pathways as a genetically influenced trait at the population level rather than a sporadic anomaly.

Rare familial studies provide complementary mechanistic insight. In a multigenerational family with structurally normal hearts, whole-exome sequencing identified a rare heterozygous missense variant in MRC2 segregating with WPW-pattern electrocardiograms and SVT, with functional modelling demonstrating inducible re-entrant tachycardia and abnormal atrioventricular insulation (21). These findings implicate extracellular matrix organisation and cell–matrix interaction pathways in maintaining annulus fibrosus integrity. Additional familial studies have prioritised variants in genes such as MYH6, suggesting that sarcomeric and myocardial structural pathways may contribute to accessory pathway susceptibility in selected families (36, 53).

Structural variation and transcriptional regulators further support a developmental model in subsets of WPW. Copy-number alterations involving the 20p12.3 region, encompassing genes such as BMP2, have been reported in association with WPW, highlighting the potential contribution of dosage-sensitive developmental pathways (54). In addition, candidate-gene association studies have implicated variants in TBX3, a transcription factor central to atrioventricular canal patterning and annulus fibrosus formation, in sporadic WPW (55). Although these findings require replication, they support disrupted atrioventricular boundary formation as a core mechanism.

Collectively, the genetic architecture of AVRT and WPW reflects a mixed model in which common variants influencing conduction and junctional development shape population risk, while rare coding variants and structural alterations can directly perturb atrioventricular insulation in selected families. Compared with other SVT subtypes, this clearer genotype–substrate relationship renders AVRT genetics more clinically actionable, supporting targeted genetic evaluation in patients with early onset, familial recurrence, associated conduction disease, or cardiomyopathic features. By contrast, the atypical non-atrioventricular accessory pathways grouped under Mahaim physiology remain primarily defined by electrophysiologic rather than genetic data, and subtype-specific genomic evidence is currently sparse (38–41).

### 5.3 Genetics of FAT

Compared with AVNRT and AVRT, the genetic basis of FAT remains poorly defined Reflecting both biological heterogeneity and methodological limitations. FAT may arise from triggered activity, enhanced automaticity, or micro-re-entry, mechanisms influenced by calcium handling, adrenergic signalling, atrial fibrosis, and localized structural abnormalities. Although these pathways are genetically modulated, reproducible gene-level or locus-level associations has not yet been demonstrated at scale.

Evidence for inherited susceptibility comes mainly from small familial and paediatric series rather than GWAS. A hereditary form of ectopic atrial tachycardia with autosomal dominant inheritance and no structural heart disease has been reported, supporting that FAT can segregate within families even when a causal gene is not identified (56). Additional early-life familial clustering has also been described in neonatal atrial tachycardia with later cardiomyopathy in an affected parent, suggesting that in some cases atrial tachyarrhythmia may represent part of a broader inherited myocardial phenotype (57).

Paediatric and foetal SVT cohorts provide indirect evidence of genetic contribution and are likely enriched for developmental and genetic determinants. In paediatric multifocal atrial tachycardia cohorts, a subset of patients later demonstrated pathogenic RYR2 variants after developing ventricular arrhythmias, and separate case reports describe RYR2 mutations presenting initially with atrial tachyarrhythmias before a more typical catecholaminergic phenotype emerges (58, 59). These findings support calcium-handling dysfunction as one potential mechanism for atrial automaticity or triggered activity in early-onset cases but do not yet define FAT-specific genetic architecture, particularly in adult populations.

FAT therefore remains a genetically underpowered phenotype and progress will require multicentre collaboration, standardized electrophysiologic phenotyping, and integration of sequencing with functional models (e.g., atrial tissue, cellular models).

### 5.4 Genetics of inappropriate sinus tachycardia (IST)

IST is a distinct supraventricular tachyarrhythmia characterized by an elevated sinus rate that is disproportionate to physiologic demand and not explained by secondary causes. Unlike AVNRT or AVRT, IST is not a re-entrant arrhythmia but is thought to reflect a combination of abnormal sinus node automaticity and autonomic dysfunction, including enhanced *β*-adrenergic responsiveness and reduced vagal restraint (60–62). From a genetic perspective, evidence remains limited. The clearest mechanistic signal comes from rare familial cases linked to gain-of-function HCN4 variants, which increase pacemaker current activity and support a biologically plausible channel-based contribution in selected patients (62–65).

However, beyond these rare familial observations, robust subtype-specific genomic associations have not been established, and IST currently remains far less genetically defined than AVNRT or accessory pathway-mediated AVRT/WPW. At present, the main relevance of including IST in an SVT genetics framework is to acknowledge it as a mechanistically distinct sinus node disorder in which heritable susceptibility is plausible, but translational application of routine genetic testing is not yet supported (60–62).

## 6 Syndromic and cardiomyopathy-associated SVT

A recurring theme in SVT genetics is mechanistic overlap with inherited cardiomyopathies and multisystem disorders, where supraventricular arrhythmias, often alongside pre-excitation or conduction disease, reflect an underlying myocardial or metabolic phenotype rather than an isolated rhythm disorder.

PRKAG2 syndrome represents the clearest example of SVT as a marker of cardiomyopathic disease. Pathogenic variants in PRKAG2, encoding the *γ*2 subunit of AMP-activated protein kinase, lead to glycogen accumulation within cardiomyocytes and progressive myocardial remodelling. Clinically, affected individuals frequently present with ventricular pre-excitation, SVT, AF, and conduction disease, often accompanied by hypertrophic cardiomyopathy, and eventual heart failure (12, 22, 66). Longitudinal cohort studies demonstrate that SVT and pre-excitation commonly precede overt cardiomyopathy or advanced conduction disease, supporting SVT as an early sentinel feature of a progressive metabolic cardiomyopathy rather than a benign arrhythmia (22).

Danon disease, caused by pathogenic variants in LAMP2, similarly highlights the prognostic importance of SVT within a syndromic framework. Paediatric and adolescent cohorts show that WPW patterns and supraventricular tachyarrhythmias are common early manifestations, often preceding severe hypertrophic or dilated cardiomyopathy and adverse outcomes including transplantation or death (67, 68). In this setting, early genetic diagnosis reframes SVT management from rhythm control alone to anticipatory care, cascade testing, and long-term planning.

Beyond metabolic and lysosomal disorders, SVT also overlaps with broader cardiomyopathy genetics, particularly in genes involved in myocardial structure and cytoskeletal integrity. Loss-of-function variants in FLNC are associated with arrhythmogenic cardiomyopathy phenotypes characterized by ventricular dysfunction, ventricular arrhythmias, and a substantial burden of supraventricular tachyarrhythmias, including SVT (69). In such conditions, supraventricular arrhythmias may coexist with or precede ventricular arrhythmias and conduction disease, serving as an early marker of disease penetrance.

Finally, rare metabolic and multisystem genetic disorders illustrate that SVT can arise secondary to systemic cellular dysfunction rather than primary electrical disease. Case reports describe SVT in conditions such as late-onset multiple acyl-CoA dehydrogenase deficiency due to ETFDH variants and in infants with ABCA3 variants, where arrhythmia occurs in the context of multisystem involvement and may improve with metabolic or supportive therapy (70, 71). Although these conditions do not define SVT genetic architecture, they highlight the importance of considering inherited metabolic disease in atypical presentation. Clinical implication: across these syndromic and cardiomyopathy-associated settings, SVT functions as a sentinel phenotype that should prompt evaluation for accompanying features such as pre-excitation, conduction disease, cardiomyopathy, skeletal muscle involvement, hepatic dysfunction, or relevant family history. In these contexts, genetic diagnosis is directly actionable, informing prognosis, cascade screening, and long-term management strategies (18, 22, 66, 68).

Key syndromic association and their clinical implications are summarised in Table 2.

**Table 2:** Syndromic and cardiomyopathy-associated SVT: genes, phenotypes, and clinical implications.

Gene/syndrome	Typical SVT-related phenotype	Accompanying features/red flags	Why diagnosis matters clinically
PRKAG2 syndrome	Pre-excitation, SVT, AF, conduction disease	Ventricular pre-excitation, hypertrophy/cardiomyopathy, progressive conduction disease, heart failure trajectory	Reframes SVT as a metabolic cardiomyopathy; guides surveillance, familial screening, and longitudinal management
Danon disease (LAMP2)	WPW-pattern/pre-excitation, supraventricular tachyarrhythmias	Severe hypertrophic/dilated cardiomyopathy, earlier adverse outcomes, multisystem disease	Early diagnosis affects prognosis, transplant planning, cascade testing, and broader anticipatory care
FLNC-related cardiomyopathy	SVT burden may coexist with ventricular arrhythmias	Ventricular dysfunction, arrhythmogenic cardiomyopathy phenotype, conduction disease	SVT may be an early marker of disease penetrance and should prompt broader inherited cardiomyopathy assessment
ETFDH-related metabolic disease	SVT reported in atypical metabolic presentations	Multisystem involvement; metabolic features	Suggests that atypical SVT context may warrant evaluation for metabolic disease
ABCA3- related disease	Infant SVT in multisystem context	Severe extracardiac illness	Highlights that SVT can occasionally be secondary to broader systemic cellular dysfunction

## 7 Molecular pathways, biomarkers, and microRNA in SVT

### 7.1 Rationale for molecular biomarkers in SVT

Genetic variation contributes to susceptibility to SVTs, but genetics alone often cannot explain arrhythmia timing, recurrence, or response to therapy because SVT expression is strongly influenced by dynamic physiological states. Molecular biomarkers offer a complementary approach by reflecting active substrate and triggers, including myocardial stretch, autonomic modulation, inflammation, and structural remodelling. This approach is supported by broader arrythmia research, where biomarkers are used to link underlying biology with electrophysiologic outcomes, although careful phenotypic and validation are required for clinical implenetation (26).

In SVT, several circulating biomarkers behave primarily as state markers rather than indicators of inherited risk. Natriuretic peptides frequently rise during paroxysmal SVT episodes and decline following rhythm normalization, consistent with acute atrial and ventricular stretch physiology (72, 73). Troponin elevation is also commonly observed in SVT presentations and is generally attributed to tachycardia related supply-demand imbalance rather than acute coronary syndromes, with recent cohort data suggesting an association with short-term adverse cardiac outcomes in selected populations (73, 74). These findings support a role for biomarkers in quantifying episode severity and short-term risk, although they do not distinguish SVT subtypes.

### 7.2 MicroRNA and exosomal signalling

MicroRNAs regulate post-transcriptional gene expression and influence pathways central to supraventricular arrhythmogenesis, including ion channel regulation, calcium handling, fibrosis, inflammation, and cellular stress responses. Although most miRNA research has focused on AF, emerging data suggest relevance to SVT. In paediatric cohorts with supraventricular arrhythmias, reduced circulating miR-1 expression has been reported in SVT compared with controls, demonstrating feasibility of miRNA-based phenotyping while highlighting heterogeneity and limited mechanistic specificity (27). Subsequent paediatric studies examining miR-1 and miR-133 family members have reported differential expression patterns across supraventricular arrhythmias, reinforcing biological plausibility but requiring electrophysiologically defined subtype stratification and replication (25).

Exosomal miRNAs offer additional translational appeal because extracellular vesicles protect RNA cargo and may better reflect myocardial biology. A recent exosomal profiling study identified miR-320c as associated with atrial fibrosis and atrial cardiomyopathy, with SVT cohorts used as comparator groups rather than the primary disease focus (24). While not SVT specific, this work demonstrates that exosome-based platforms can capture atrial substrate signals potentially relevant to SVT risk and recurrence. Mechanistic studies further support plausibility by showing that cardiomyocyte derived exosomal miRNAs can promote fibroblast activation and extracellular matrix remodelling, processes central to atrial substrate development (75).

Overall, current miRNA and exosome signatures reflect generalized atrial remodelling rather than discrete SVT mechanisms, highlighting the need for precise electrophysiologic phenotyping in future studies.

### 7.3 Signalling pathways and cellular stress responses

Beyond miRNAs, several non-electrical cellular pathways plausibly shape the substrate permissive for SVT, including TGF-*β* related profibrotic signalling, inflammatory cascades, oxidative stress, and apoptosis–autophagy balance. These processes can alter connexin expression, tissue architecture, and calcium handling, thereby influencing susceptibility to re-entry or triggered activity. Although SVT specific mechanistic studies remain limited, pathway level insights from atrial arrhythmia research provide a rational framework for hypothesis driven biomarker discovery and functional validation in SVT relevant models (76).

Emerging clinical studies have begun to explore accessible inflammatory and stress related markers in SVT populations. Elevated red cell distribution width and other systemic indices have been reported in SVT cohorts, suggesting an association between inflammatory or stress related states and supraventricular arrhythmias, although these findings remain preliminary and not subtype resolved (77). Overall, the most coherent translational strategy is to integrate molecular biomarkers with precise electrophysiologic phenotyping and clinically meaningful outcomes, rather than extrapolating AF biomarker paradigms directly to SVT.

## 8 Functional validation and translational models

A central challenge in SVT genetics is the gap between statistical association and mechanistic causality. This limitation is particularly pronounced because the key substrates underlying SVT, including the atrioventricular node and atrioventricular junction, are anatomically small, regionally specialized, and defined by tissue level conduction properties that are difficult to reproduce in simplified experimental systems. As a result, many genetic associations cannot yet be linked with confidence to specific electrophysiological mechanisms such as nodal duality, slow pathway conduction, or accessory pathway mediated re-entry.

Human induced pluripotent stem cell derived cardiomyocytes have become foundational tools for functional genomics in inherited arrhythmias, enabling scalable interrogation of variant effects on ion channels and calcium handling. However, conventional atrial or ventricular cardiomyocyte models do not recapitulate atrioventricular nodal biology. Recent advances in directed differentiation, three-dimensional engineered tissues or organoid systems are beginning to generate atrioventricular conduction axis-like cell states and spatial organization, offering new opportunities to model nodal and peri-annular physiology relevant to AVNRT and AVRT (78, 79). These platforms aim to bridge genetic discovery with mechanism-informed intervention, while emphasizing the need for SVT-specific readouts rather than extrapolation from ventricular arrhythmia models.

Animal models remain essential for studying SVT mechanisms because re-entry depends on intact three-dimensional architecture, atrioventricular insulation, and realistic cell-to-cell coupling. Developmental mouse models disrupting atrioventricular canal patterning, including perturbations of Tbx2 and related pathways, have demonstrated how annulus fibrosus malformation leads to fast conducting accessory pathways and ventricular pre-excitation, providing direct genotype-to-substrate links relevant to AVRT (35, 37). In parallel, models of PRKAG2 related cardiomyopathy illustrate how metabolic remodelling can secondarily disrupt atrioventricular insulation and promote pre-excitation phenotypes (80). Integration of these models with human genetic data is essential for establishing causal mechanisms in conduction-related arrhythmias.

Progress in SVT genetics will depend on integrated translational pipelines linking human genetic discovery with functional models capable of interrogating nodal conduction, atrioventricular insulation, and re-entry at appropriate biological scales. Scalable and reproducible systems that connect genotype to electrophysiologic mechanism are essential to move beyond association and enable clinically actionable insights.

Relevant molecular biomarkers and translational models are summarised in Table 3.

**Table 3:** Molecular biomarkers and translational models relevant to SVT.

Category	Examples discussed in manuscript	What they may reflect	Main limitation	Current role
Acute circulating biomarkers	Natriuretic peptides, troponin	Episode-related myocardial stretch, haemodynamic stress, supply-demand imbalance	Mostly state markers rather than inherited-risk markers; poor subtype specificity	Potential adjuncts for episode severity and short-term risk assessment
Circulating microRNAs	miR-1, miR-133 family	Ion channel regulation, calcium handling, fibrosis, cellular stress	Small studies, paediatric-heavy data, heterogeneity, limited mechanistic specificity	Early biomarker signal; not ready for routine clinical use
Exosomal miRNAs	miR-320c	Atrial fibrosis/atrial cardiomyopathy-related biology	Not SVT-specific; reflects generalised atrial remodelling more than discrete SVT mechanism	Translationally promising but investigational
Inflammatory/stress markers	RDW and related systemic indices	Inflammatory or stress-related arrhythmia milieu	Preliminary, not subtype resolved	Hypothesis-generating only
Functional cellular models	hiPSC-derived cardiomyocytes, emerging AV canal-like/organoid systems	Variant-level functional effects; conduction biology modelling	Standard iPSC atrial/ventricular models do not fully recapitulate AV nodal tissue	Essential research tools for moving from association to mechanism
Animal models	Developmental AV canal/annulus fibrosus models; PRKAG2-relaed models	AV insulation, re-entry substrate, genotype-to-phenotype validation	Translational gap between animal model and human phenotype	Critical for causal interference, especially in AVRT/WPW and syndromic SVT

## 9 Genetic testing in SVT: diagnostic yield, cost-effectiveness, and clinical integration

### 9.1 Diagnostic yield of genetic testing in SVT and related arrhythmia syndromes

The diagnostic yield of genetic testing in SVT depends strongly on whether the presentation reflects a largely polygenic common phenotype or a monogenic cardiogenetic disorder in which SVT is part of a broader syndrome. In unselected patients with typical AVNRT or AVRT and a structurally normal heart, broad panel testing generally has low yield and limited actionability because susceptibility appears to be largely polygenic, with common-variant association signals that are informative for biology but not yet positioned for routine diagnostic use (14). In contrast, diagnostic yield increases meaningfully when SVT is accompanied by clinical “red flags” suggesting a monogenic substrate, such as ventricular pre-excitation with hypertrophy, conduction system disease, early onset disease, a family history of cardiomyopathy or sudden death, or extracardiac features. In these settings, phenotype-guided cardiogenetic evaluation with structured variant interpretation increases diagnostic yield and reduces misinterpretation of variants of uncertain significance (18, 81).

Syndromic and cardiomyopathy-associated SVT provides the clearest high-yield scenario. Disorders that produce pre-excitation and SVT alongside myocardial disease, including PRKAG2 syndrome and Danon disease (LAMP2), are classically actionable because a genetic diagnosis clarifies prognosis, guides surveillance and family screening, and reframes SVT as a marker of systemic or progressive cardiac disease rather than an isolated electrophysiological problem (12, 82).These examples highlight the need for a stratified approach, as identical ECG phenotypes may reflect either benign accessory pathway physiology or underlying cardiomyopathy where genetic diagnosis alters management.

### 9.2 Cost-effectiveness and health economic considerations

Economic value in inherited arrhythmia genotyping is determined less by sequencing cost and more by downstream clinical consequences, including prevention of adverse events, avoidance of low-yield investigations, and identification of at-risk relatives through cascade testing (17). Real-world costing studies support this principle. In a Canadian inherited heart rhythm disorder referral pathway, comprehensive evaluation and testing generated substantial costs, while cascade testing in relatives was notably less expensive per diagnosis than testing in primary referrals, reinforcing the economic logic of phenotype-guided proband testing followed by targeted family testing when a pathogenic variant is identified (83). Health-system assessments similarly emphasize that pathway design, counselling capacity, and downstream investigations are major cost drivers and therefore central to sustainability, particularly when testing is applied broadly outside high-pretest-probability settings (84–86).

Applied to SVT, these data support a stratified approaching which genetic testing is reserved for presentations where results are likely to alter prognosis, surveillance, or family management, rather than routine panel testing in isolated, typical AVNRT or AVRT in structurally normal hearts.

### 9.3 Implementation pathways and clinical governance

Clinical integration of genetics into SVT care requires governance structures that minimize harm from uncertain findings and maximize utility when a diagnosis is actionable. Recent expert consensus and laboratory quality recommendations converge on several principles including careful patient selection, pre-test counselling, targeted test design, rigorous variant interpretation, and post-test follow-up within multidisciplinary teams that integrate electrophysiology, imaging, family history, and genetics expertise (81, 87). This is particularly important in SVT because testing low-pretest-probability cases increases the likelihood of variants of uncertain significance and may generate anxiety, unnecessary cascade testing, or inappropriate changes in management without clear benefit (81).

Accordingly, SVT genetics is best embedded within inherited arrhythmia or cardiogenetic services rather than managed in isolation, with referral thresholds informed by phenotype and comorbidity context. This model aligns diagnostic yield, economic value, and patient safety by reserving broad testing for higher-likelihood presentations while enabling rapid targeted testing and cascade screening when a monogenic diagnosis is suspected or confirmed (18, 88).

## 10 Genotype-guided therapy and implications for pharmacological and ablation strategies

### 10.1 Pharmacological therapy in the context of SVT Genetics

For most patients with typical AVNRT or AVRT and structurally normal hearts, pharmacological therapy is used for acute termination or symptom suppression while catheter ablation remains definitive. In this common SVT setting, there is currently no established role for genotype-guided drug selection, largely because actionable genotype drug interaction data are lacking and the therapeutic ceiling for improvement is limited by the high efficacy of ablation (1).

Genetic context becomes clinically important when SVT occurs within inherited arrhythmia syndromes or cardiomyopathy overlap states. In Brugada syndrome, supraventricular tachyarrhythmias, particularly AF and other SVTs, are clinically relevant because they can precipitate inappropriate ICD therapies and complicate risk management, making rhythm control strategies and drug selection contingent on the underlying syndrome rather than the SVT mechanism alone (89, 90). In these patients, the practical implication is often not a specific genotype matched drug, but genotype-informed avoidance of proarrhythmic exposures and earlier consideration of non-pharmacologic rhythm control when drug options are constrained by syndrome-specific risk (91, 92).

Genetic context also informs pharmacological risk in pre-excitation mediated phenotypes. In pre-excited atrial fibrillation, AV nodal blocking agents can be harmful because they may facilitate preferential conduction via an accessory pathway, and guideline-directed management emphasizes rhythm conversion strategies appropriate to this physiology rather than routine AV nodal blockade (1). This distinction is particularly important when pre-excitation reflects an underlying cardiogenetic condition rather than an isolated electrophysiological variant, emphasising how genetics can guide medication safety through refined phenotype classification (1).

In metabolic and lysosomal cardiomyopathies associated with pre-excitation and SVT, genetics has direct therapeutic implications because conduction disease and progressive myocardial dysfunction are part of the expected trajectory. In PRKAG2 syndrome, ventricular pre-excitation, SVT, hypertrophy, and progressive conduction disease frequently coexist, so chronic AV nodal blocking therapy may worsen bradyarrhythmias or contribute to hemodynamic compromise in susceptible individuals, requiring management strategies that address the underlying syndrome rather than SVT suppression alone (12, 66, 93). Similarly, Danon disease is strongly associated with hypertrophic cardiomyopathy and pre-excitation, and early genetic recognition supports anticipatory care and family screening, with SVT serving as a potential early marker of a broader high-risk phenotype (67, 94).

### 10.2 Catheter ablation outcomes and genetic context

Catheter ablation is highly effective for AVNRT and AVRT, and in most patients with structurally normal hearts genetic information does not alter procedural strategy or expected acute success. Contemporary guideline recommendations therefore position ablation as definitive therapy based primarily on clinical presentation and electrophysiologic diagnosis rather than genotype (1).

Genetic context becomes relevant to procedural timing, follow-up intensity, and broader risk framing in selected subgroups. In syndromic pre-excitation cardiomyopathies such as PRKAG2 syndrome, ablation may address the tachycardia mechanism, but progressive conduction disease and cardiomyopathy can still shape longitudinal management and outcomes, so the procedural endpoint should not be interpreted as completion of disease management (12, 66).

Genotype-guided ablation in common SVT remains investigational, as validated genetic predictors of recurrence or substrate complexity are not yet available for routine use. The clearest current clinical utility of genetics is indirect, supporting identifying syndromic contexts in which drug choices, risk counselling, device management considerations, and longitudinal surveillance differ materially from isolated AVNRT or AVRT (81, 91).

## 11 Emerging gene therapies and molecular interventions

Gene based therapeutics represent the most forward-looking intersection of genetics and arrhythmia management. Over the last several years, inherited arrhythmia research has moved from conceptual discussions of feasibility toward increasingly mature preclinical platforms spanning adeno-associated virus (AAV) mediated gene replacement, allele specific silencing, and genome editing, with a small but growing number of programs progressing into early human studies in cardiomyopathy and related electrical phenotypes (95–97). In cardiac channelopathies, preclinical work increasingly includes disease-targeted strategies such as gene replacement for LQT1, with KCNQ1 augmentation demonstrating correction of QT prolongation and cellular electrophysiology abnormalities in transgenic rabbit models (98). Parallel progress in CPVT has been supported by multiple AAV-based gene therapy approaches, including CASQ2 replacement strategies, with evidence supporting biological efficacy alongside ongoing challenged related to delivery and long-term safety (99, 100).

These advances are relevant to SVT genetics primarily as a translational framework rather than as near-term SVT specific therapy. SVT substrates such as AV nodal re-entry and accessory pathway mediated re-entry are anatomically localized and circuit based, making direct gene therapy aimed at modifying re-entrant pathways unlikely in the near term. The more plausible near-term intersection is indirect, where syndromic SVT occurs as part of a molecular cardiomyopathy in which correcting the underlying defect could improve the myocardial milieu that sustains arrhythmia and conduction disease. Danon disease provides the clearest example that cardiomyopathy gene therapy has moved into early clinical evaluation. Early phase human studies of AAV9 LAMP2B gene therapy have reported clinical and biomarker endpoints consistent with biological activity, alongside detailed attention to immune responses and safety monitoring that will be instructive for future cardiac gene therapy programs (101). PRKAG2 syndrome is also discussed in translational gene therapy reviews and has preclinical genome editing proof of concept in postnatal models, reinforcing the idea that cardiomyopathy associated pre-excitation phenotypes may ultimately be approached through disease correction rather than SVT circuit targeting (100).

In parallel, RNA based therapeutics are emerging as another clinically realistic route to molecular intervention in arrhythmogenic disease. Antisense oligonucleotides have advanced rapidly in cardiovascular genetics, and proof of principle has been demonstrated for arrhythmia relevant conditions such as calmodulinopathy, supporting allele specific silencing concepts that may be generalizable to other rare inherited arrhythmia syndromes (102). Conceptually, miRNA modulation remains attractive for atrial remodelling biology, but translation to SVT will require stronger causal pathway definition and far greater delivery specificity, particularly if targets involve conduction tissue or AV nodal physiology rather than diffuse atrial substrate (44, 103).

Key barriers include delivery specificity, off-target effects, durability, and safety, particularly in conduction tissue where small perturbations may carry disproportionate electrophysiologic consequences. For SVT, the most defensible clinical relevance in the near term is therefore gene and RNA therapies developed for cardiomyopathy and inherited arrhythmia syndromes may modify SVT burden and risk trajectories in syndromic disease, while establishing the technical and governance frameworks required for future SVT-specific applications (101, 104, 105).

## 12 Ethical, economic, and implementation considerations

The expansion of clinical genomics into SVT care raises ethical challenges that differ in important ways from those encountered in malignant inherited arrhythmia syndromes. Informed consent must address the likelihood of uncertain or incidental findings, the potential for reinterpretation over time, and the implications of results for biological relatives. These issues are particularly salient in SVT, where variants of uncertain significance are common and where the clinical actionability of many findings remains limited, increasing the risk of overinterpretation and harm if results are not contextualized appropriately (81, 106).

A central ethical tension in SVT genomics relates to proportionality. Many SVT phenotypes are benign and highly treatable, yet genetic testing can reframe patient identity around inherited disease risk, with downstream consequences for anxiety, insurance, and family dynamics. This contrasts with conditions where genomic testing is clearly lifesaving and underscores the importance of reserving testing for contexts where results are likely to alter management, prognosis, or family screening strategies (107). Cascade testing further amplifies these considerations, as relatives may be exposed to uncertainty or surveillance burdens without clear benefit when pathogenicity or penetrance is unclear.

Economic considerations extend well beyond the cost of sequencing itself. Real-world evaluations of inherited arrhythmia services consistently show that downstream investigations, longitudinal surveillance, multidisciplinary staffing, and counselling infrastructure are the dominant cost drivers, rather than test pricing alone (88, 108). These findings are directly relevant to SVT, where indiscriminate testing in low-yield populations risks generating sustained system costs without commensurate clinical benefit. Health-services research further demonstrates that pathway design, including referral criteria, triage processes, and access to genetics expertise, largely determines both cost-effectiveness and clinical utility (85, 86).

Equity of access represents an additional implementation challenge. Specialized cardiogenetic services are unevenly distributed across healthcare systems, and patients with isolated SVT are less likely to be referred for expert genomic evaluation than those with overt cardiomyopathy or malignant arrhythmia phenotypes. Without deliberate implementation planning, expansion of SVT genomics risks amplifying disparities based on geography, socioeconomic status, and health literacy (88, 107). Models embedding genetics within existing electrophysiology or inherited arrhythmia services appear most effective in balancing access, expertise, and governance.

Finally, the psychological impact of genetic labelling in SVT should not be underestimated. Qualitative and cohort studies in cardiogenetics consistently show that uncertain or non-actionable results can increase anxiety and alter illness perception, particularly when patients previously understood their condition as benign and curable (107, 109). Careful pre-test counselling, clear communication of limitations, and structured post-test follow-up are therefore essential to ensure that genomic information empowers patients without causing unintended psychological or social harm.

Practical approaches to clinical integration of genetics in SVT are summarised in Table 4.

**Table 4:** Practical clinical integration of genetics in SVT.

Clinical scenario	Expected yield/utility of genetic testing	Suggested approach	Rationale
Typical isolated AVNRT or AVRT in a structurally normal heart	Low diagnostic yield; limited immediate actionability	Do not perform indiscriminate broad testing routinely	Most common SVT appears largely polygenic; testing risks uncertain findings without changing clinical management
Early-onset or familial SVT	Higher potential relevance	Consider phenotype-guided genetics referral, especially if recurrence Is familial or phenotype is unusual	Raises suspicion for inherited substrate beyond sporadic disease
SVT with extracardiac features or strong family history of cardiomyopathy/sudden death	Higher likelihood of actionable diagnosis	Multidisciplinary evaluation with imaging, family history review, and phenotype-directed testing	Increase pre-test probability and reduces misinterpretation
Low-test-probability testing without phenotypic enrichment	Poor value	Avoid	Increases VUS burden, anxiety, downstream costs, and potentially unnecessary cascade testing
Confirmed monogenic diagnosis	High utility	Cascade testing, syndrome-specific surveillance, tailored counselling	This is where genetics most clearly changes prognosis and familial management

## 13 Discussion

This narrative review synthesizes molecular, clinical, and translational evidence supporting a meaningful genetic contribution to SVT susceptibility and expression. Collectively, the data challenge the long-held perception of SVT as a purely functional or incidental arrhythmia and instead position it along a continuum of inherited cardiac electrophysiological disorders (11, 13).

Several themes emerge. First, genetic architecture differs substantially by SVT subtype. AVNRT exhibits a polygenic and oligogenic pattern, with common variants in developmental and structural genes shaping nodal substrate permissiveness. AVRT and WPW represent the most genetically interpretable SVT phenotypes, reflecting their developmental origin and strong associations with both common and rare variants. Focal atrial tachycardia remains genetically underexplored, highlighting an urgent need for better phenotyping and collaborative research (13). Supporting evidence from familial case series and systematic synthesis reinforces that AVNRT heritability is detectable but complex, while nationwide familial WPW recurrence studies add epidemiological weight to accessory pathway heritability (11–13).

Second, the greatest current clinical utility of SVT genetics lies in recognizing syndromic and cardiomyopathy-associated disease. In PRKAG2 and LAMP2 disease, SVT is a gateway to diagnosis, prognostication, and family screening, demonstrating how genetics can transform care when effect sizes are large and mechanisms are clear (66, 68, 80). This stands in contrast to common SVT, where genetics primarily informs biology rather than immediate management. The growing cardiomyopathy genetics literature also supports SVT as an early or accompanying feature in selected inherited cardiomyopathies, including arrhythmogenic cardiomyopathy-related genotypes (69, 110).

Third, translational advances in functional genomics, biomarker discovery, and gene therapy provide tools to bridge association and mechanism. microRNA and exosomal platforms illustrate how molecular signatures may complement genetic risk, while advanced *in vitro* and animal models offer pathways to validate candidate genes and pathways. However, these approaches must be tightly coupled to electrophysiological phenotype to avoid dilution of signal (13, 111). Pathway-level work implicating TGF-*β*/SMAD signalling, apoptosis, autophagy, and inflammatory remodelling may be particularly relevant to substrate formation across supraventricular phenotypes, including FAT and atrial remodelling adjacent to accessory pathway substrates (76, 112).

Finally, implementation science is as critical as discovery. Diagnostic yield, cost-effectiveness, ethical governance, and health system integration will determine whether SVT genetics advances from academic insight to routine care. The experience of inherited arrhythmia clinics provides a blueprint, but SVT-specific pathways must be developed thoughtfully (15, 17, 88, 107).

## 14 Future directions

Future progress in SVT genetics will depend on coordinated advances across discovery, functional validation, and clinical implementation, informed by recent SVT-specific genetic and translational developments. On the discovery side, emerging GWAS have now identified reproducible loci associated with common SVT phenotypes, including AVNRT and accessory pathway–mediated tachycardia, underscoring the feasibility of genetic discovery when large, well-phenotyped cohorts are used (13, 20). These findings highlight the importance of standardized electrophysiological phenotyping and support integrating genomic data with intermediate traits such as conduction parameters, ECG features, autonomic responsiveness, and molecular biomarkers to improve mechanistic interpretability and predictive value (13, 113, 114).

Functional validation must evolve in parallel to address SVT-specific substrates. Recent advances in human stem cell biology now enable generation of atrioventricular canal–like cardiomyocytes and engineered organoid systems that reproduce key features of atrioventricular conduction, representing a major step toward modelling nodal and peri-annular physiology *in vitro* (79, 80). Together with high-resolution mapping in genetically defined animal models, these platforms provide a realistic pathway to link candidate variants to conduction properties and re-entry mechanisms relevant to AVNRT and AVRT. Ensuring that such systems are scalable, reproducible, and quantitatively electrophysiologic remains essential for translational relevance (14, 115).

Clinically, prospective studies are now needed to determine whether genetic information meaningfully alters management decisions, outcomes, and healthcare utilization in SVT. Given the generally low diagnostic yield in unselected SVT populations, such studies should focus on phenotype-enriched cohorts and explicitly evaluate downstream consequences, including changes in ablation strategy, surveillance, family screening, and patient-reported outcomes (88, 107). Recent implementation and governance guidance in inherited cardiac disease further emphasizes that pathway design, counselling infrastructure, and proportional use of testing will be critical determinants of clinical utility and equity as SVT genetics moves toward broader adoption (15, 85, 107).

## 15 Conclusions

Genetic predictors of supraventricular tachycardia are reshaping our understanding of these common arrhythmias. While routine genetic testing is not yet indicated for most patients with typical SVT, substantial evidence supports inherited contributions to susceptibility, particularly for AVNRT and AVRT, and unequivocal clinical value exists in syndromic and cardiomyopathy-associated SVT. By integrating genetic discovery with functional validation, biomarker development, and implementation science, the field is poised to move toward more precise and anticipatory SVT care. Continued multidisciplinary collaboration will be essential to translate genetic insight into tangible benefits for patients and families.
